# BATF3-dependent dendritic cells drive both effector and regulatory T-cell responses in bacterially infected tissues

**DOI:** 10.1371/journal.ppat.1007866

**Published:** 2019-06-12

**Authors:** Isabelle C. Arnold, Xiaozhou Zhang, Mariela Artola-Boran, Angela Fallegger, Peter Sander, Pål Johansen, Anne Müller

**Affiliations:** 1 Institute of Molecular Cancer Research, University of Zurich, Zurich, Switzerland; 2 Institute of Medical Microbiology, University of Zurich, Zurich, Switzerland; 3 Department of Dermatology, University of Zurich and University Hospital Zurich, Zurich, Switzerland; Stanford University School of Medicine, UNITED STATES

## Abstract

The gastric lamina propria of mice that have been experimentally infected with the pathobiont *Helicobacter pylori* hosts a dense network of myeloid cells that includes BATF3-dependent CD103^+^ dendritic cells (DCs). We show here that CD103^+^ DCs are strictly required for gastric Th1 responses to *H*. *pylori* and for *H*. *pylori* infection control. A similar dependence of type 1 immunity on CD103^+^ DCs is observed in a *Mycobacterium bovis* BCG infection model, and in a syngeneic colon cancer model. Strikingly, we find that not only the expansion and/or recruitment of Th1 cells, but also of peripherally induced, neuropilin-negative regulatory T-cells to sites of infection requires BATF3-dependent DCs. A shared feature of the examined models is the strongly reduced production of the chemokines and CXCR3 ligands CXCL9, 10 and 11 in BATF3-deficient mice. The results implicate BATF3-dependent DCs in the recruitment of CXCR3^+^ effector and regulatory T-cells to target tissues and in their local expansion.

## Author summary

In this work, Arnold & Zhang et al report that CD103^+^ DCs are required for protective Th1 responses, infection control of mucosal and systemic bacterial pathogens, and anti-tumor immunity driven by CD4^+^ Th1 cells and CD8^+^ T cells. CD103^+^ DCs further specifically promote the recruitment of Tbet^+^ peripherally induced Tregs to sites of infection. The results implicate CD103^+^ DCs in the trafficking of CXCR3^+^ Tbet^+^ T-cells to sites of infection and tumorigenesis.

## Introduction

Mononuclear phagocytes (MPs) residing in the lamina propria (LP) of the gastrointestinal (GI) tract comprise populations of resident macrophages that are replenished from Ly6C^hi^ blood monocytes, and of dendritic cells (DCs) that originate from a pre-DC-progenitor and require FLT3L for their development [1, 2]. Three gastrointestinal DC lineages exist in the steady state; these can be identified based on their surface marker expression, dependence on transcription and growth factors, and functional specialization. CD103^+^CD11b^-^ DCs require the basic leucine zipper transcription factor ATF-like 3 (BATF3) and interferon regulatory factor 8 (IRF8) for their development [3] and are equipped to cross-present viral, tumor, and self-antigens; the functional human equivalent of CD103^+^CD11b^-^ DCs is the CD141^hi^ DC subset [4]. The development of CD103^+^CD11b^+^ DCs depends on granulocyte macrophage colony-stimulating factor (GM-CSF) [5, 6] as well as the transcription factors Notch-2 [7] and IRF4 [8]. Functionally, IRF4/Notch-2-dependent DCs (likely human equivalent: CD1c^+^ DCs) have been implicated in Th17 priming [7, 8]. The third DC subset of the GI tract expresses CD11b and intermediate levels of CX_3_CR1, but neither CD103 nor typical macrophage markers such as F4/80, Ly6C, or CD64. This DC population shares the IRF4/Notch-2 dependence of CD103^+^CD11b^+^ DCs and is responsive to FLT3L *in vivo*, but its progenitor is controversial [9–11]. The lymph node counterparts of the described LP DCs are CD11b^+^ and CD8α^+^ DCs, which share the reliance on IRF4/Notch-2 and IRF8/BATF3, respectively.

We have recently conducted a detailed analysis of gastric LP DC populations in the steady state and during infection with the gastric pathobiont *Helicobacter pylori* and were able to confirm the existence of all three intestinal DC subsets also in this organ, albeit at frequencies that differed from the intestines [12]. CD11b^+^CX_3_CR1^int^ DCs represented the dominant gastric DC population and exhibited properties of monocytes/macrophages, i.e. CX_3_CR1 expression and the cell-intrinsic dependence on CCR2 signaling, as well as the FLT3L dependence of DCs [12]. CD11b^+^CX_3_CR1^int^ DCs further showed a previously unknown developmental requirement for the inflammasome sensor NLRP3, but not other inflammasome components, a dependence that applied also to CD11b^+^CX_3_CR1^int^ DCs of other GI tissues and the lung [12]. CD103^+^CD11b^+^ DCs were also detected in the stomach, especially upon infection with *H*. *pylori*, and were present at low frequencies that resembled those of the colon rather than those of the small intestine, where they represent the most dominant DC population by far. CD103^+^CD11b^-^ DCs were recruited to the *H*. *pylori*-infected stomach along with monocytes, CX_3_CR1^hi^ macrophages and CD11b^+^ and CD103b^+^CD11b^+^ DCs, but, in contrast to the former populations, appear not to encounter live bacteria in the gastric LP of mice infected with red fluorescent protein-expressing (RFP^+^) *H*. *pylori* [12].

We and others have reported that DCs that have been exposed to live *H*. *pylori* acquire tolerogenic properties that drive regulatory T-cell (Treg) rather than T-effector responses [13–18]. The ability of *H*. *pylori* to manipulate DC functionality is particularly evident in an experimental mouse model that entails neonatal infection with *H*. *pylori* [19] and mimics the natural maternal-to-offspring transmission of *H*. *pylori* that is characteristic of human populations in which *H*. *pylori* is endemic [20]. In children and in neonatally infected mice, the *H*. *pylori*-host interaction is generally asymptomatic and characterized by the lack of effector T-cell responses, high level colonization and predominance of Tregs [19, 21]. In contrast, *H*. *pylori*-infected adults, especially those presenting with peptic ulcers, and mice infected as adults, exhibit a T-cell infiltrate that limits the bacterial burden without clearing *H*. *pylori* completely and is dominated by effector T-cells [19, 21]. The mutualism that is characteristic of the interaction between *H*. *pylori* and its (neonatally infected) host has been linked to secondary protective effects against allergic asthma, other forms of allergy, and against inflammatory bowel diseases in humans [22–24] and in mice [15, 25, 26].

Here, we address the functional contribution of BATF3-dependent CD103^+^ DCs to *H*. *pylori*-specific immunity and immune tolerance. We examine the role of BATF3-dependent CD103^+^ DCs in models of mucosal and systemic bacterial infection and investigate how BATF3-dependent DCs contribute to T-cell-driven immunity to bacteria and to anti-tumor immunity. Our data reveal a non-redundant role of CD103^+^ DCs in promoting both effector T-cell and regulatory T-cell responses in bacterially infected tissues, which we attribute to their ability to recruit and/or locally expand Tbet^+^ T-cell populations.

## Results

### CD103^+^ DCs drive type I immunity to a mucosal bacterial infection

**Fig 1:**
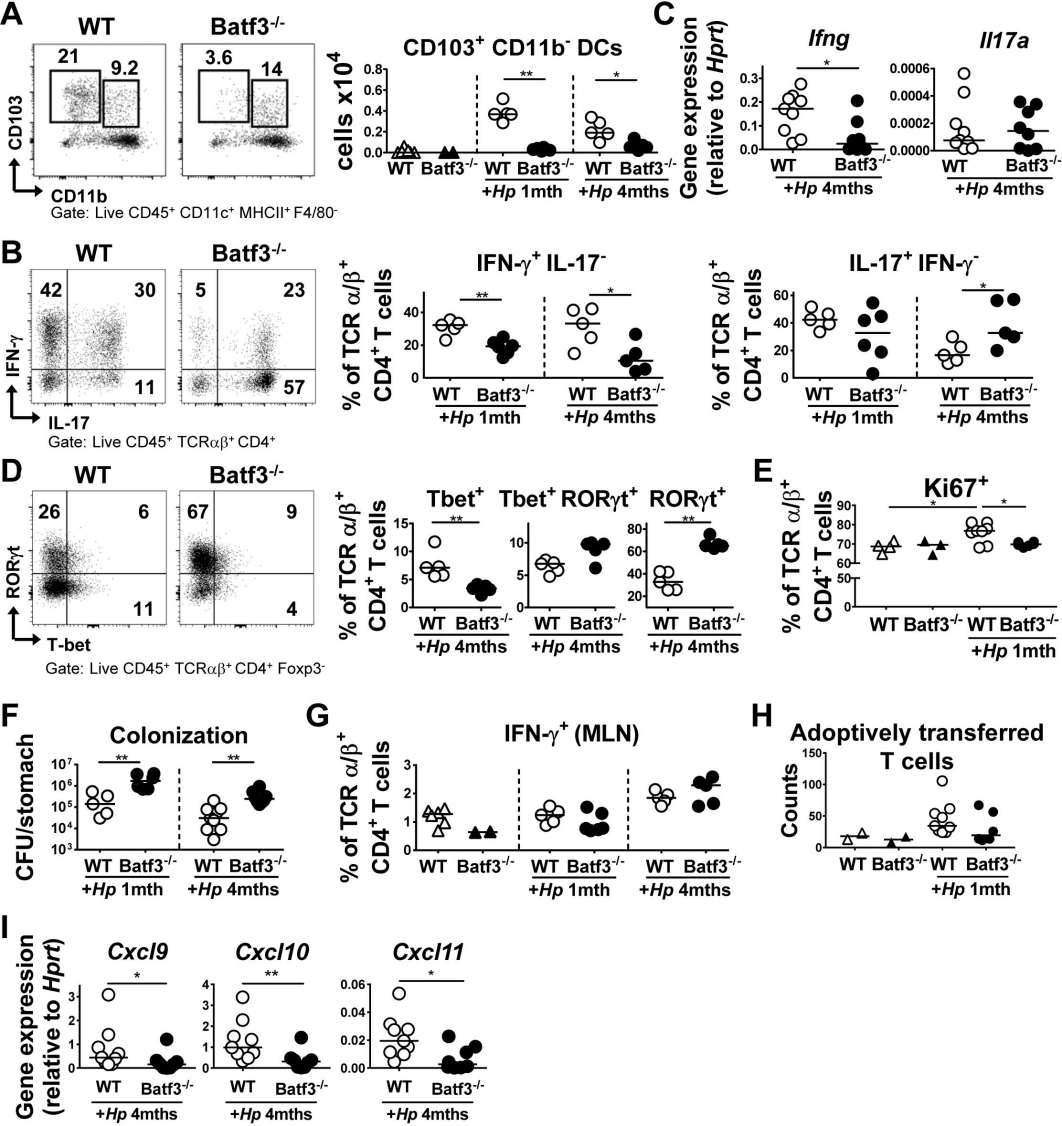
BATF3-dependent DCs are required for local Th1 responses and bacterial infection control. (A-D) BATF3^-/-^ and WT mice were infected with *H*. *pylori* and their gastric LP leukocytes were analyzed by FACS at one or four months p.i. (A) Absolute counts of CD103^+^ CD11b^-^ DCs in the gastric LP of naïve and infected WT and BATF3^-/-^ mice. Representative FACS plots are shown alongside summary plots. (B) Frequencies of IFN-γ^+^ and IL-17^+^ CD4^+^ T-cells of the mice shown in A. The summary plot shows a representative time course of three independently conducted experiments. (C) Expression of IFN-γ and IL-17 in the gastric mucosa of WT and BATF3^-/-^ mice at four months p.i.; results are pooled from two independent experiments. (D) RORγt and Tbet expression of LP CD4^+^ T-cells of the mice shown in A at four months p.i. (E) Mice of the two genotypes were infected as described above, and subjected to Ki67 staining of their LP T-cells. Results are from one experiment. (F) *H*. *pylori* colonization of the mice shown in A-D. (G) IFN-γ expression of CD4^+^ T-cells in the MLNs of WT and BATF3^-/-^ mice infected with *H*. *pylori* for 1 and/or 4 months as shown in A. Results are representative of two independent experiments. (H) Absolute numbers of adoptively transferred CD45.1^+^ CD4^+^ T-cells recovered from the gastric LP of recipients of the indicated genotypes five days post adoptive transfer. Naïve recipients of T-cells are shown as controls alongside *H*. *pylori*-infected recipients. (I) Expression of CXCL9, 10 and 11 in the gastric mucosa of WT and BATF3^-/-^ mice four months p.i.; results are pooled from two independent experiments.

Having observed that CD103^+^ DCs are recruited to the *H*. *pylori*-infected gastric mucosa [12], we examined their functional contribution to *H*. *pylori*-specific immunity in mice that lack the transcription factor BATF3. The gastric LP of BATF3^-/-^ animals exhibited strongly reduced numbers of CD103^+^CD11b^-^ DCs, but was populated by normal complements of CD103^+^CD11b^+^ DCs, D11b^+^ DCs and macrophages, both in the steady state and during infection (Fig 1A, S1A Fig). Interestingly, BATF3^-/-^ mice had reduced local Th1 responses upon infection as determined by intracellular cytokine staining for IFN-γ and by quantification of IFN-γ transcript levels in the gastric mucosa (Fig 1B and 1C). In contrast, Th17 responses were normal at one month post infection (p.i.), and even enhanced at 4 months p.i. (Fig 1B and 1C). The reduced Th1 but not Th17 response in infected BATF3^-/-^ mice was reflected by reduced frequencies of Tbet^+^, but not of RORγt^+^ CD4^+^ T-cells in the infected gastric mucosa (Fig 1D). The absolute numbers of cytokine-expressing cells per organ, and of Tbet^+^/RORγt^+^ CD4^+^ T-cells per organ, confirmed the trends observed for the frequencies of the respective populations (S1B and S1C Fig). We further found a lower frequency of actively cycling, Ki67^+^ T-cells in the mucosa of BATF3^-/-^ relative to WT mice (Fig 1E, S1D Fig). As a likely consequence of their inability to generate strong gastric Th1 responses, BATF3^-/-^ mice exhibited a clear defect in controlling an *H*. *pylori* infection (Fig 1F). To rule out that possible differences in the microbiota composition of WT and BATF3^-/-^ mice drive the observed differences in *H*. *pylori*-specific T-cell responses, mice of both genotypes were co-housed from birth to the end-point of a four week *H*. *pylori* infection (i.e. for a total of ten weeks). The exact same results as before were obtained under these experimental conditions: we observed significant hyper-colonisation of BATF3^-/-^ mice, accompanied by a strong defect in generating Th1 responses (S1E and S1F Fig). The phenotype of BATF3^-/-^ mice could not be attributed to a defect in Th1 priming, as the frequencies of IFN-γ-expressing Th1 cells in the draining mesenteric lymph nodes (MLNs) were normal in BATF3^-/-^ mice (Fig 1G). The phenotype of BATF3^-/-^ mice further could also not be linked to differences present already at steady state, as Th1 populations were indistinguishable in the absence of an *H*. *pylori* infection (S1F Fig). To rule out that the failure of BATF3^-/-^ mice to recruit T-cells to, or activate them in the infected gastric mucosa was T-cell intrinsic, we adoptively transferred CD45.1^+^ cells that we had isolated from the spleens of *H*. *pylori*-infected donors into WT or BATF3^-/-^ recipients that had been infected for four weeks with *H*. *pylori*. Although very few adoptively transferred cells could be re-isolated from the recipients after five days, their recruitment to the gastric mucosa appeared to be infection- and also somewhat BATF3-dependent (Fig 1H).

Tbet-positive T-cells express the chemokine receptor CXCR3, which allows the cells to migrate along gradients of the CXCR3 ligands CXCL9, CXCL10 and CXCL11 to their target tissues [27, 28]. We therefore examined the expression of these chemokines in *H*. *pylori*-infected gastric tissue. Interestingly, the gastric mucosa of BATF3^-/-^ mice exhibited lower expression levels of CXCL9, CXCL10 and CXCL11 than that of their WT counterparts (Fig 1I). To identify the DC population(s) that could serve as major sources of these chemokines in the gastric lamina propria, and to examine the relative contribution of the three chemokines, we flow cytometrically sorted CD103^+^, CD11b^+^ and CD103^+^CD11b^+^ DCs from the lamina propria of infected and uninfected WT mice and subjected pools of 30–100 highly pure cells to qRT-PCR analysis. Whereas CXCL11 transcripts were extremely rare in all three populations, the other two chemokines were clearly detectable in all three DC subsets; exposure to *H*. *pylori* resulted in enhanced expression of CXCL10, whereas CXCL9 was expressed constitutively (S1G Fig). The combined results suggest that CD103^+^ DCs have a non-redundant role in providing the local signals driving Th1 recruitment and expansion and bacterial infection control in this mucosal infection model; however, this contribution cannot be attributed to CD103^+^ DCs being the exclusive source of these chemokines in the gastric lamina propria.

### CD103^+^ DCs promote Th1 responses to systemic infection with *M*. *bovis* BCG

**Fig 2:**
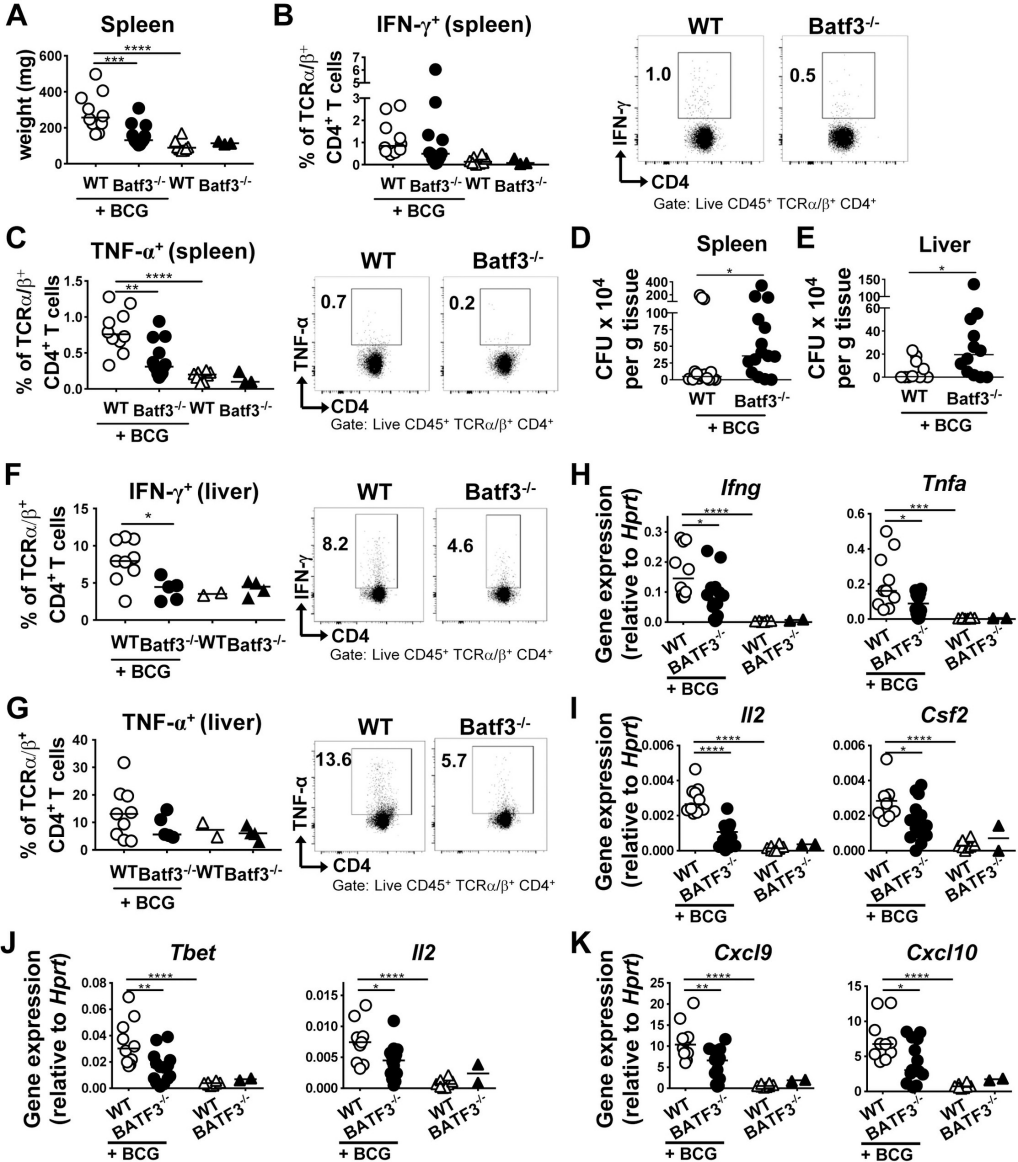
BATF3-dependent DCs are required for systemic bacterial infection control. (A-K) BATF3^-/-^ and WT mice were infected with *M*. *bovis* BCG for three weeks and their spleen weights (A), splenic frequencies of IFN-γ^+^ CD4^+^ T-cells (B, summary plot and representative FACS plots), splenic frequencies of TNF-α^+^ CD4^+^ T-cells (C), and splenic BCG colonization (D) are shown. Restimulation of T-cells was performed with BCG-specific purified protein derivate (PPD). Pooled data from two independent experiments are shown throughout with the exception of panel D, which is from three pooled experiments. (E-K) Liver colonization of mice infected with BCG as described above (E), Th1 responses (F,G) and gene expression of transcripts associated with Th1 polarization (H-J) and with chemokine production (K), as assessed by qRT-PCR and normalized to *Hprt*. Data in E and H-K are pooled from two studies; data in F and G are from one study and representative of two independent ones.

To assess whether BATF3-dependent DCs also contribute to the control of systemic bacterial infection, we infected WT and BATF3-deficient mice with *M*. *bovis* BCG. At three weeks p.i., spleens were isolated for the analysis of bacterial growth and of BCG-specific immune responses. The spleens and livers of BATF3^-/-^ mice were lighter than WT spleens (Fig 2A), exhibited reduced Th1 responses as determined by intracellular cytokine staining for IFN-γ and TNF-α (Fig 2B and 2C), and were colonized at higher levels than WT spleens (Fig 2D). Similar observations with respect to colonization and the magnitude of BCG-specific Th1 responses were made in the liver, which in BATF3^-/-^ mice was colonized at higher levels than in WT mice (Fig 2E) and which, just like the spleen, exhibited reduced Th1 responses (Fig 2F and 2G). Th17 responses were not elevated due to BCG infection of the liver, and did not differ between the two strains (S2A Fig). We also observed clear BATF3-dependent differences in infection-induced cytokine expression in the liver. The Th1 cytokines IFN-γ, TNF-α, GM-CSF and IL-2 were all produced at higher levels in WT relative to BATF3^-/-^ mice (Fig 2H and 2I). A similar pattern was observed for the Th1-specific transcription factor Tbet and the Th1-polarizing cytokine IL-12 (Fig 2J). In contrast, the expression of cytokines and transcription factors associated with Th2-, Th17-, and regulatory T-cell responses was similar in the livers of infected WT and BATF3^-/-^ mice (S2B Fig). Interestingly, and as observed after infection of mice with *H*. *pylori*, the expression of the chemokines CXCL9 and CXCL10 was strongly reduced in BCG-infected BATF3-deficient compared to WT mice (Fig 2K), whereas CXCL11 expression was unaffected by the BCG infection or the mouse genotype (S2B Fig). The combined results suggest that the (Th1-dependent) control of mucosal as well as systemic bacterial infections requires BATF3-dependent DCs.

### CD103^+^ DCs support anti-tumor immunity by promoting Th1 and CD8^+^ T-cell responses

**Fig 3:**
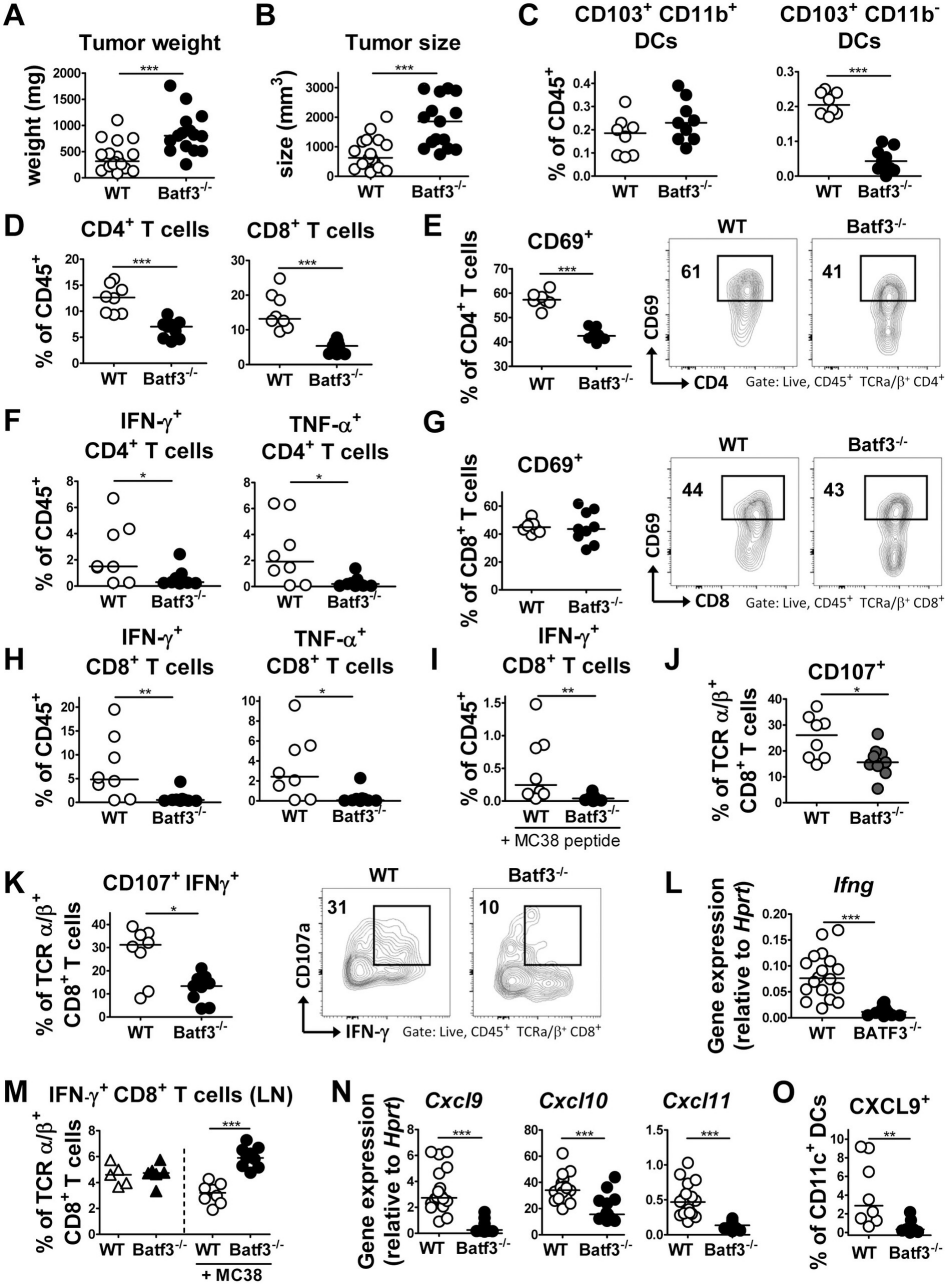
BATF3-dependent DCs are required for CD4^+^ and CD8^+^ T-cell recruitment to the tumor microenvironment and for tumor control. (A-O) WT and BATF3^-/-^ mice were subcutaneously injected in both flanks with 5×10^5^ MC38 cells. Tumors were analyzed after 15 days with respect to tumor weight (A), volume (B), leukocyte infiltration (C-K, M, and O) and mRNA expression profile (L and N). (A,B) Tumor weights and volumes as determined at the study endpoint. Data are pooled from two independent studies. (C,D) Frequencies of CD103^+^ CD11b^+^ and CD103^+^ CD11b^-^ DCs (C) and of CD4^+^ and CD8^+^ T-cells (D) among all tumor-infiltrating leukocytes. One representative study of two is shown. (E) Frequencies of CD69^+^ CD4^+^ T-cells. Data are from the study shown in C and D, and presented along with representative FACS plots. (F) Frequencies of IFN-γ^+^ and TNF-α^+^ CD4^+^ T-cells among all CD45^+^ T-cells, as assessed upon re-stimulation with PMA/ionomycin. Data are from the study shown in C and D, and representative of two independent ones. (G) Frequencies of CD69^+^ CD8^+^ T-cells. Data are from the study shown in C and D, and presented along with representative FACS plots. (H) Frequencies of IFN-γ^+^ and TNF-α^+^ CD8^+^ T-cells among all CD45^+^ T-cells, as assessed upon re-stimulation with PMA/ionomycin. Data are from the study shown in C and D, and representative of two independent ones. (I) Frequencies of IFN-γ^+^ CD8^+^ T-cells among all CD45^+^ T-cells, as assessed upon re-stimulation with MC38 tumor-specific peptide. (J) Frequencies of CD107^+^ CD8^+^ T-cells. Data are from the study shown in C and D, and presented along with representative FACS plots. (K) Frequencies of CD8^+^ T-cells co-expressing CD107 and IFN-γ. Data are from the study shown in C and D, and presented along with representative FACS plots. (L) IFN-γ expression as assessed by qRT-PCR of tumor tissue, of the studies shown in A and B. (M) Frequencies of IFN-γ^+^ CD8^+^ T-cells in the inguinal LNs of tumor-bearing (+MC38) relative to naïve mice, as assessed upon re-stimulation with PMA/ionomycin. Data are from the study shown in C and D, and representative of two independent ones. (N) Expression of the indicated T-cell-attracting chemokines, as assessed by qRT-PCR of tumor tissue, of the studies shown in A and B. Horizontal lines indicate medians throughout; statistical analyses were done using the Mann-Whitney test. *p<0.05; **p<0.01; ***p<0.0005. (O) Frequencies of CXCL9^+^ DCs among all CD11c^+^ cells. Data are from the study shown in C and D; see S3I Fig for the gating strategy and representative FACS plots.

To investigate the role of BATF3-dependent DCs in a model of T-cell driven anti-tumor immunity, we took advantage of the highly immunogenic MC38 colon cancer xenograft model, which is known to be strictly controlled by both CD4^+^ and CD8^+^ T-cells. We found that BATF3 deficiency facilitated MC38 tumor growth (Fig 3A and 3B). MC38 tumors were infiltrated by a variety of immune cells, of which CD103^+^ DCs constituted a small but robust population, both in terms of their frequencies and absolute numbers (Fig 3C, S3A Fig). As expected, BATF3-dependent CD103^+^CD11b^-^ DCs were absent in tumors of BATF3^-/-^ mice, whereas CD103^+^CD11b^+^ DCs infiltrated the tumors of BATF3-proficient and -deficient mice at similar frequencies and absolute numbers (Fig 3C, S3A Fig). Interestingly, the infiltration of CD4^+^ and CD8^+^ T-cells, but not of NK cells, into the tumor mass depended strongly on BATF3-dependent DCs (Fig 3D, S3B and S3C Fig). The residual population of tumor-infiltrating CD4^+^ T-cells exhibited reduced expression of the early activation marker CD69 (Fig 3E). Th1 cells, identified by their expression of IFN-γ and TNF-α, were reduced both in frequencies and numbers in the tumor mass of BATF3^-/-^ mice (Fig 3F, S3D Fig). CD8^+^ T-cells in tumors from WT and BATF3-deficient mice did not differ in terms of their CD69 expression (Fig 3G); there were however much fewer cytokine-expressing CD8^+^ T-cells detectable in tumors of BATF3^-/-^ relative to WT mice, as assessed both upon re-stimulation with PMA/ionomycin, as well as with MC38 tumor antigen-derived peptide (Fig 3H and 3I, S3E Fig). CD69 expression on tumor-associated NK cells was similar in WT and BATF3-deficient mice (S3F Fig). Interestingly, the cytotoxic activity of CD8^+^ T-cells, as judged by expression of their degranulation marker CD107 and their granzyme B expression, was strongly reduced in the absence of BATF3-dependent DCs (Fig 3J, S3G Fig). Intratumoral frequencies of multifunctional CD8^+^ T-cells expressing both cytokines and the degranulation marker CD107 were particularly strongly reduced in BATF3^-/-^ mice (Fig 3K). These results were confirmed by qRT-PCR analysis of IFN-γ and TNF-α expression performed on tumor tissues (Fig 3L, S3H Fig). Interestingly, the defect of BATF3^-/-^ mice in generating anti-tumor immune responses could not be attributed to a priming defect, as frequencies of cytokine-producing CD8^+^ T-cells were higher rather than lower in the draining inguinal lymph nodes of tumor-bearing BATF3^-/-^ mice relative to WT controls (Fig 3M). Rather, tumors in BATF3^-/-^ mice lacked expression of the T-cell recruiting chemokines CXCL9, CXCL10, and CXCL11 (Fig 3N), which could be attributed to differential CXCL9 expression in the DC compartment (Fig 3O, S3I Fig). In summary, in the absence of this DC subset, T-cell priming appears to occur normally but activated T-cells fail to migrate to the tumor site due to lack of the appropriate chemokine gradient.

### CD103^+^ DCs promote the recruitment of Tbet^+^ RORγt^+^ peripherally induced Tregs to sites of infection

**Fig 4:**
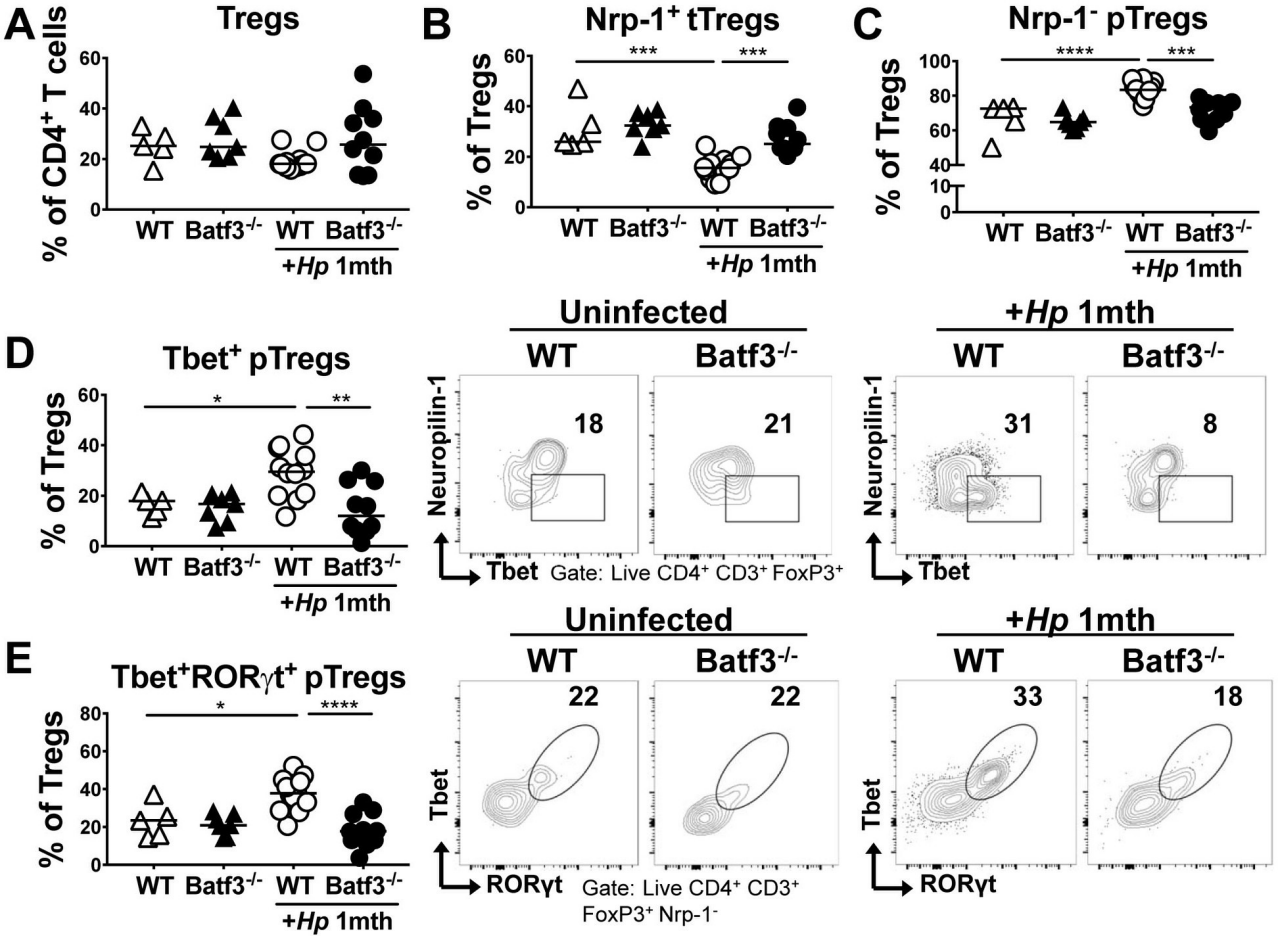
BATF3-dependent DCs are required for peripherally induced Treg recruitment to the LP of the GI tract. (A-E) WT and BATF3^-/-^ mice were infected with *H*. *pylori* and the gastric LP Treg compartment was analyzed one month p.i. by FACS and compared to that of naïve controls of both genotypes. (A) Frequencies of LP Foxp3^+^ regulatory CD4^+^ T-cells. Data are pooled from two independent experiments, and representative of four experiments. (B,C) Frequencies of neuropilin-1 (Nrp-1)-positive thymus-derived and Nrp-1-negative peripherally-induced Tregs (tTregs, pTregs) among all CD4^+^ Foxp3^+^ LP Tregs. Data are from the studies shown in A. (D,E) Frequencies of Tbet^+^ (D) and Tbet^+^RORγt^+^ (E) pTregs among all CD4^+^ Foxp3^+^ LP Tregs. Summary and representative FACS plots are shown. Horizontal lines indicate medians throughout; statistical analyses were done using one-way ANOVA followed by Holm-Sidak’s multiple comparisons correction. *p<0.05; **p<0.01; ***p<0.0005; ****p<0.0001.

We have shown previously that BATF3-dependent DCs are required for immune tolerance to *H*. *pylori* and for the immunomodulatory effects of *H*. *pylori* in models of allergen-induced asthma [29], which in turn are driven by *H*. *pylori*-induced, highly suppressive regulatory T-cells (Tregs) [15, 25, 30]. To address whether BATF3-dependent DCs are required for the *H*. *pylori-*dependent differentiation and recruitment of Tregs, we infected WT and BATF3^-/-^ mice with *H*. *pylori* for one month (as before, Fig 1) and analyzed the gastric LP Treg compartment by flow cytometry. Although we found the frequencies of CD4^+^ Foxp3^+^ Tregs to be comparable in the gastric LP of WT and BATF3^-/-^ mice, both in the steady state and upon infection (Fig 4A), the composition of the Treg compartment differed substantially as a result of BATF3 deficiency. We observed a shift from thymus-derived, neuropilin-positive tTregs to peripherally induced, neuropilin-negative pTregs in the WT gastric LP as a consequence of *H*. *pylori* infection (Fig 4B and 4C); pTregs were largely Tbet- and RORγt-positive (Fig 4D and 4E). The recruitment of pTregs was substantially reduced in the gastric LP of BATF3^-/-^ mice and this observation was particularly evident for Tbet^+^ RORγt^+^ pTregs (Fig 4B–4E); the changes in Treg subset frequencies were mirrored by the absolute counts of the same populations (suppl Fig 4A–4E). Other Treg or general T-cell activation markers (i.e. TIGIT, CD44, Tim3) did not vary measurably due to BATF3 deficiency (suppl Fig 4F–4H). The defective pTreg recruitment to the site of infection in BATF3^-/-^ mice could not be attributed to a differential composition of the microbiota of WT and BATF3^-/-^ mice, as co-housing the mice from birth until the study endpoint produced the same results, i.e. a selective defect in the recruitment of pTregs expressing Tbet and RORγt to the site of infection (suppl Fig 4I–4M). The combined results suggest that BATF3^-/-^ mice exhibit a specific defect in the recruitment of pTregs to target tissues.

### BATF3^-/-^ mice fail to induce CXCR3 expression in peripherally induced Tregs

**Fig 5:**
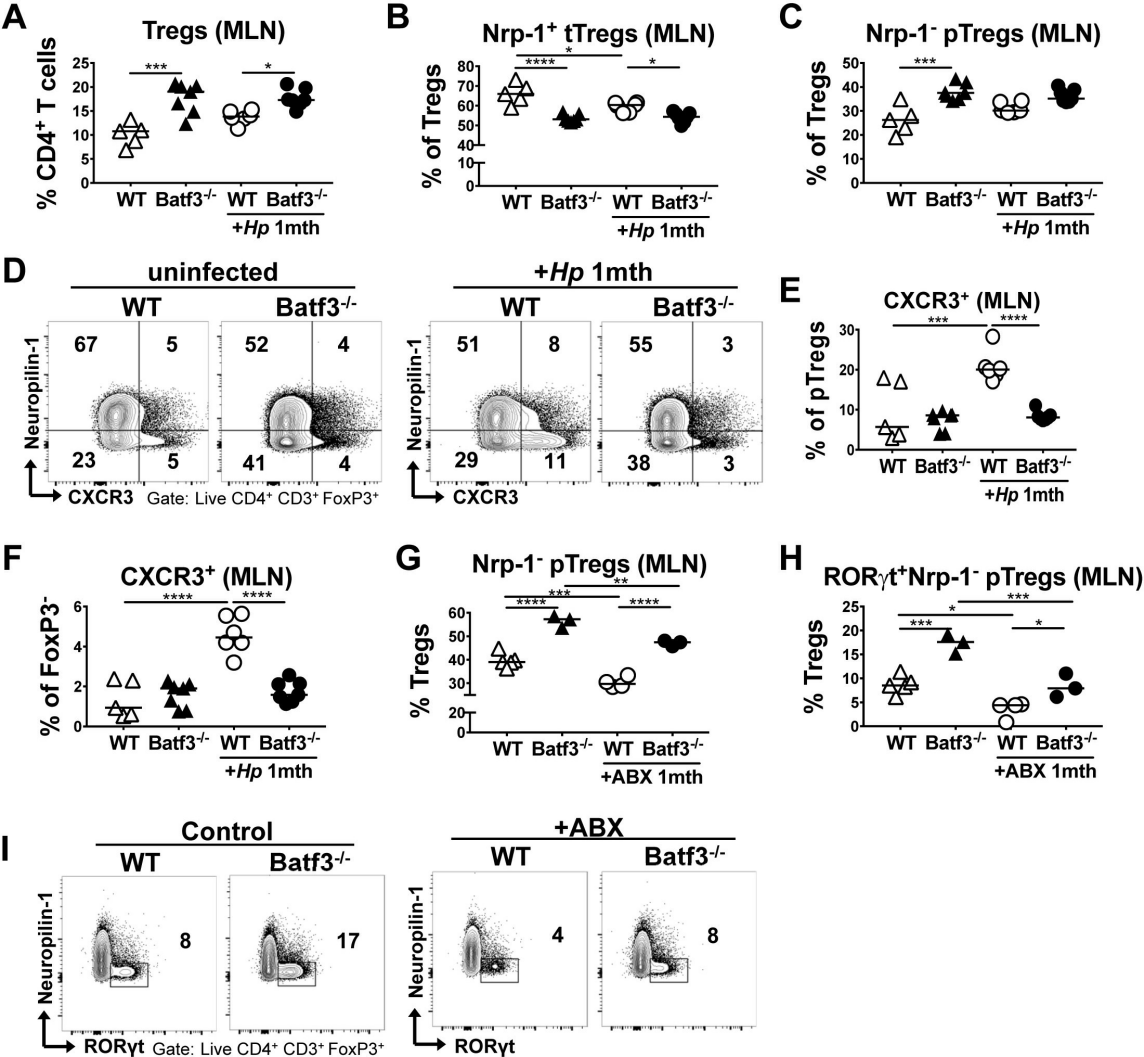
BATF3-dependent DCs are required for the induction of CXCR3 expression on peripherally induced Tregs in the MLNs. (A-F) WT and BATF3^-/-^ mice were infected with *H*. *pylori* and the MLN Treg compartment was analyzed one month p.i. by FACS and compared to that of naïve controls of both genotypes. MLN frequencies of all FoxP3^+^ Tregs (A), tTregs (B) and pTregs (C) are shown, along with the frequencies of CXCR3^+^ pTregs and their representative FACS plots (D,E) and of CXCR3^+^ CD4^+^ Foxp3^-^ T-cells (F). (G-I) WT and BATF3^-/-^ mice were subjected to a four week-long course of antibiotic eradication therapy prior to the analysis of their MLN Treg compartment by FACS. MLN frequencies of Foxp3^+^ Nrp^-^ pTregs (G), and RORγt^+^ pTregs (H) are shown relative to untreated controls, and alongside representative FACS plots in I. Horizontal lines indicate medians throughout and p-values were calculated using one-way ANOVA followed by Holm-Sidak’s multiple comparisons correction. *p<0.05; **p<0.01; ***p<0.0005; ****p<0.0001.

We next addressed whether the differences in pTreg recruitment to the site of infection in WT and BATF3^-/-^ mice were attributable to differential priming of Tregs in the MLNs upon *H*. *pylori* infection. Interestingly, we found (p)Treg populations to be elevated rather than reduced in the MLNs of BATF3^-/-^ mice, as assessed in terms of their frequencies (Fig 5A–5C) and absolute numbers (suppl Fig 5A–5C). This effect was also observed if animals were co-housed (suppl Fig 5D–5F). Our analyses thus corroborate earlier reports of an over-representation of Foxp3^+^ Tregs in the LNs of BATF3-deficient mice [31]. As we could not attribute the defect of pTreg recruitment to the infected stomach to a generalized deficiency in the pTreg compartment, we focused on the CXCR3 expression of MLN Tregs as an indicator of their ability to traffic to infected tissues along chemokine gradients. Most CXCR3-expressing Tregs in the MLNs of infected mice were neuropilin-negative pTregs (Fig 5D) that were also positive for RORγt. The frequency of this population in the MLNs increased strongly upon infection, and this was highly dependent on BATF3 (Fig 5E). The same differential expression of CXCR3 was observed in Foxp3^-^ CD4^+^ effector T-cells of the same MLN preparations (Fig 5F). To examine using an independent, complementary approach whether neuropilin-negative pTregs are indeed peripherally and microbially induced, we subjected mice to a four week course of antibiotic eradication using a combination of the four antibiotics ampicillin, vancomycin, neomycin and metronidazole, administered via the drinking water. Eradicated mice exhibited significantly reduced frequencies of neuropilin-negative pTregs, and especially of neuropilin-negative RORγt-positive pTregs in the MLNs (Fig 5G–5I), and this reduction in pTregs was observed irrespective of BATF3 proficiency. The combined results attribute the pTreg recruitment defect of BATF3^-/-^ mice to their inability to induce the expression of the chemokine receptor CXCR3 on pTregs during their priming in lymph nodes on the one hand, and their inability to generate gradients of the chemokine ligands of this receptor, CXCL9, 10 and 11, in infected tissues on the other.

### The neutralization of CXCR3 phenocopies some but not all consequences of BATF3 deficiency

**Fig 6:**
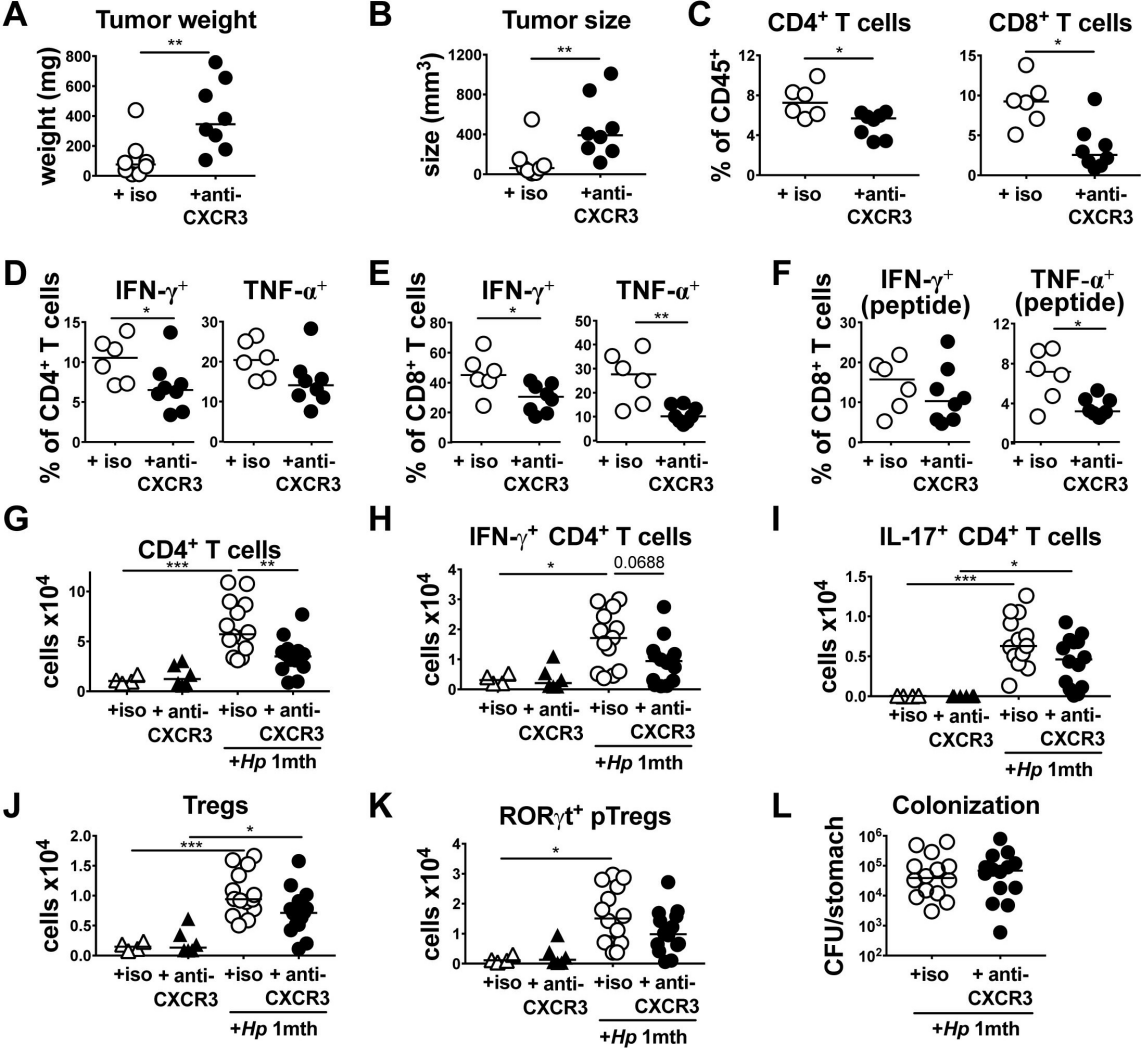
CXCR3 signaling is required for type I immunity and tumor control. (A-F) WT mice were subcutaneously injected in both flanks with 5×10^5^ MC38 cells and received twice weekly doses of CXCR3-neutralizing or isotype control antibody from the day of tumor cell injection. Tumors were analyzed after 15 days with respect to tumor weight, volume and T-cell infiltration. (A,B) Tumor weights and volumes as determined at the study endpoint. (C) Frequencies of CD4^+^ and CD8^+^ T-cells among all tumor-infiltrating leukocytes. (D-F) Frequencies of IFN-γ^+^ and TNF-α^+^ CD4^+^ and CD8^+^ T-cells, as assessed upon re-stimulation with PMA/ionomycin (D,E) or upon re-stimulation with MC38 peptide (F). (G-L) WT mice were infected for four weeks with *H*. *pylori* and received twice weekly doses of CXCR3-neutralizing or isotype control antibody starting from the day of infection. (G) Absolute counts of CD4^+^ T-cells per stomach. (H,I) Absolute counts of IFN-γ^+^ and IL-17^+^ CD4^+^ T-cells per stomach. (J,K) Absolute counts of Foxp3^+^ Tregs and of Foxp3^+^ Nrp^-^ pTregs per stomach. (L) *H*. *pylori* colonization of the mice shown in G-K. Data in A-F are from one experiment and data from G-L are pooled from two independent ones. Horizontal lines indicate medians throughout and p-values were calculated using by Mann-Whitney test (A-F) and one-way ANOVA followed by Holm-Sidak’s multiple comparisons correction (G-L).

The dual defect of BATF3^-/-^ mice in priming Tregs and effector T-cells that express CXCR3 and in generating chemokine gradients for T-cell recruitment to infected tissues provided a strong rationale for blocking the interaction of CXCR3 with its ligands with a neutralizing antibody in the MC38 tumor model and during *H*. *pylori* infection. In the MC38 tumor model, twice weekly intraperitoneal administration of a CXCR3-neutralizing antibody led to larger and heavier tumors at the study endpoint (Fig 6A and 6B), a strongly reduced infiltration of CD4^+^ and of CD8^+^ T-cells into the tumor mass (Fig 6C) and reduced IFN-γ and TNF-α production by the residual populations of both CD4^+^ and CD8^+^ T-cells (Fig 6D–6F). Similarly, CXCR3 neutralization during a four week time course of *H*. *pylori* infection strongly reduced the infiltration of all CD4^+^ T-cells and of Th1 cells into the infected gastric mucosa (Fig 6G and 6H). Trends toward lower Th17 cells and Foxp3^+^ Tregs, as well as lower RORgt^+^ pTregs were observed in CXCR3 antibody-treated mice (Fig 6I–6K). *H*. *pylori* colonization levels did not differ significantly as a consequence of CXCR3 neutralization (Fig 6L). The combined results lend further support to the idea that the CXCL9,10/CXCR3 signaling axis is required for Th1 and Treg trafficking to target tissues.

### CD103^+^ DCs promote the systemic immunomodulatory effects of *H*. *pylori* infection

**Fig 7:**
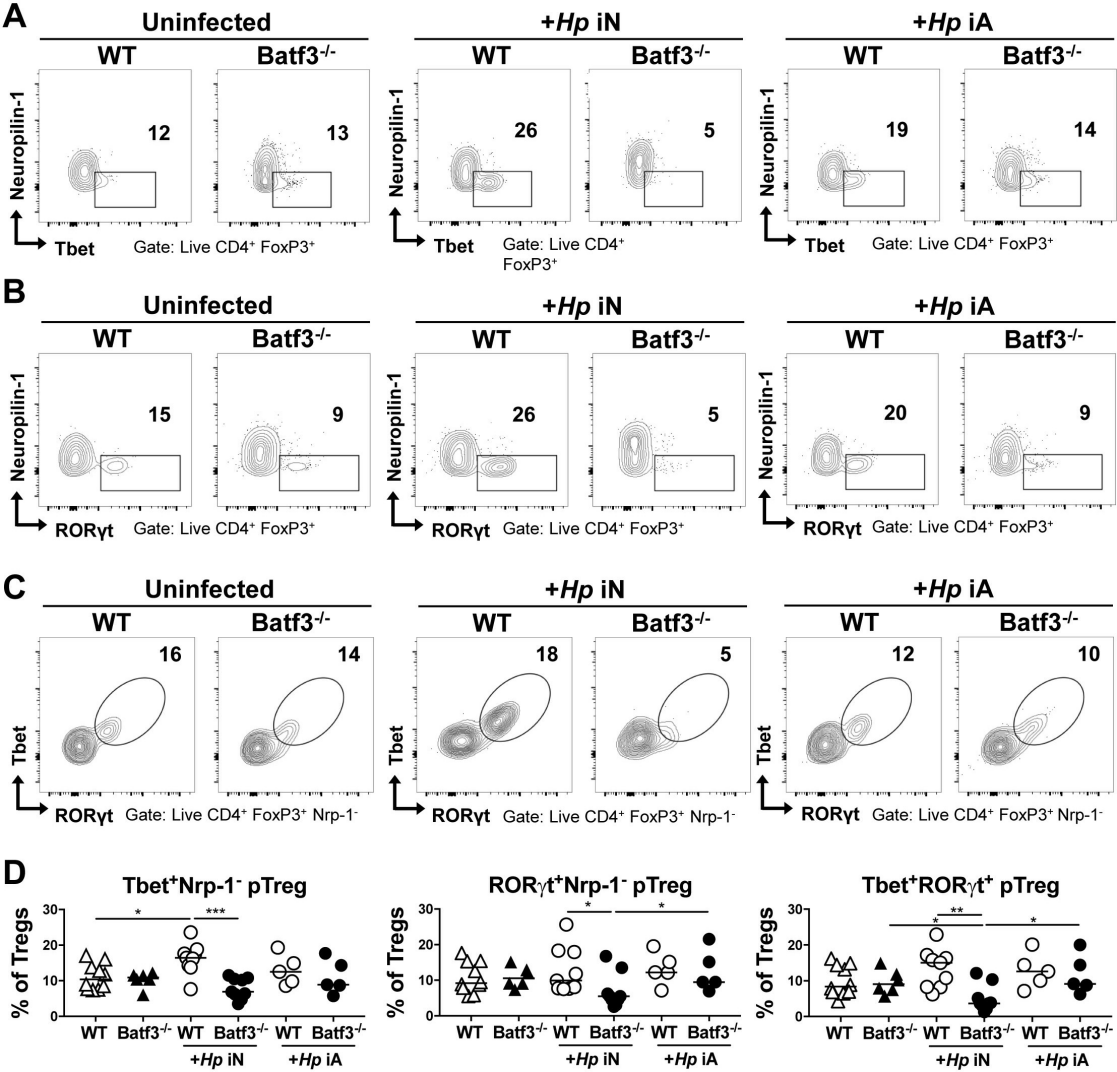
BATF3-dependent DCs promote the recruitment of Tbet^+^ RORγt^+^ pTregs to the lungs of neonatally infected mice. (A-D) BATF3^-/-^ and WT mice were infected with *H*. *pylori* either as neonates (iN) or as adults (iA), and the lungs were analyzed by FACS 6 (iN) and 4 (iA) weeks p.i. (A) Representative FACS plots of neuropilin vs. Tbet expression. (B) Representative FACS plots of neuropilin vs. RORγt expression. (C) Representative FACS plots of Tbet vs. RORγt expression. (D) Frequencies of the pTreg subsets shown in A-C among all pulmonary Foxp3^+^ Tregs. In all graphs, horizontal lines indicate medians and p-values were calculated using ANOVA. *p<0.05; **p<0.01; ***p<0.0005. Data in A-D are pooled from two experiments, and are representative of three independently conducted ones.

As BATF3-dependent DCs are required for immune tolerance to *H*. *pylori* and for the immunomodulatory effects of *H*. *pylori* in models of allergen-induced airway inflammation [29], we examined the frequencies of pulmonary Tregs in WT and BATF3^-/-^ mice that were infected with *H*. *pylori*. As only mice that have been experimentally infected in the neonatal period benefit from reduced allergy symptoms whereas mice infected as adults do not [25], we compared pulmonary Treg populations of mice infected neonatally and as adults. We observed a modest but reproducible increase in the frequencies of neuropilin-negative pTregs expressing Tbet and RORγt as a consequence of neonatal, but not adult infection in WT mice; this increase was dependent on BATF3 (Fig 7A–7D). Tbet and RORγt were co-expressed in pulmonary pTregs (Fig 7C). Overall, the changes in Treg subsets in the pulmonary compartment are consistent with our previous observation of lack of immune tolerance to airway inflammation in BATF3^-/-^ mice and implicate microbially induced pTregs originating in the GI tract in immune regulation at distant sites.

## Discussion

We show here that BATF3-dependent DCs are absolutely required for the Th1-driven control of a mucosal and a systemic bacterial pathogen, *H*. *pylori* and *M*. *bovis* BCG. Our data are in line with previous evidence for a critical role of BATF3-dependent CD103^+^ DCs in the activation and expansion of Th1 cells in murine models of *Leishmania major* infection [32] and in immune protection against the newly identified protist *Tritrichomonas musculis* that colonizes the murine intestine and triggers strongly Th1-polarized T-cell responses [33]. Our data support a model where BATF3-dependent DCs are dispensable for T-cell priming -as we find Th1 cell frequencies in the MLNs to be relatively normal- but indispensible for Th1 cell recruitment to, and expansion in the target tissue. BATF3-dependent CD103^+^ DCs of the GI tract are better known for their potency at driving CD8^+^ T cell immunity [3, 34, 35] and for their role in priming Treg differentiation through the production of retinoic acid [36, 37]. Our data, as well as the results from the *Leishmania* and *Tritrichomonas musculis* studies [32], further attribute an essential function in promoting local Th1 responses to this versatile DC subset in the GI tract.

In addition to their role in promoting type I immunity to bacterial infections, CD103^+^ DCs are indispensable for tumor control. CD103^+^ DCs are found at low but consistent frequencies in the tumor microenvironment of highly immunogenic tumor cell lines such as the MC38 colon carcinoma cell line used here. Their specific loss leads to larger tumors that exhibit reduced infiltration of both CD4^+^ and CD8^+^ T-cells, despite the fact that T-cell priming appears to be normal in tumor-bearing BATF3^-/-^ mice. Our results are consistent with previous reports that have linked functional CD103^+^ DCs to therapy success in models of cancer immunotherapy with anti-CD137 and anti-PD-1 antibodies [38], of adoptive T-cell transfer therapy [39] and even of chemotherapy [40]. In these experimental models, CD103^+^ DCs were shown to be required for cross-priming of tumor antigens [38] and for T-cell trafficking of adoptively transferred therapeutic T-cells [39], respectively. The success of chemotherapy was enhanced by anti-TIM3 antibodies that presumably targeted the CD103^+^ DC compartment in this setting [40]. In several of these therapeutic scenarios, intratumoral CD103^+^ DCs contributed to tumor immunity through production of T-cell recruiting chemokines such as CXCL9 and CXCL10 [39, 40]. We show here that BATF3-dependent CD103^+^ DCs are not only required for therapy success as reported previously, but also for the spontaneous control of tumor growth. Our data point to a dual role of CD103^+^ DCs in T-cell trafficking: in the absence of this DC subset, chemokine gradients that in WT mice would allow T-cells to migrate to infected tissues or tumors are not established, and the expression of the chemokine receptor for CXCL9 and CXCL10, CXCR3, is not induced during priming in the draining LNs.

To our surprise, we found that CD103^+^ DCs are not only required for effector T-cell recruitment and function in tumor and infected tissues, but also for pTreg recruitment to sites of infection. The gastric LP Treg compartment in naive mice consists of roughly equally sized populations of neuropilin-positive thymus-derived tTregs and peripherally induced pTregs. This distribution is different from that found in the intestines, where pTregs comprise ~80% of the total Treg pool [41]. Upon infection with *H*. *pylori*, the pTreg compartment expands at the expense of tTregs, and a significant fraction of the gastric LP pTreg population that is generated during infection expresses Tbet and RORγt. The increase in gastric LP pTreg frequencies upon infection can largely be attributed to an increase in these Tbet^+^ RORγt^+^ pTregs, and is highly dependent on BATF3. Previous reports have shown that the differentiation of pTregs in the periphery requires microbial signals (germ-free mice for example lack this population) as well as TGF-β and retinoic acid [42, 43]. CD103^+^ DCs are a well-known source of both factors in the gut LP [36, 44]; activation of the TGF-β signaling pathway in naïve T-cells leads to the binding of SMAD2 and SMAD3 to a response element, termed conserved non-coding sequence 1 (CNS1), which is part of a *Foxp3* intronic enhancer and is known to regulate Foxp3 expression in pTregs [45]. CNS1 also contains a binding site for the retinoic acid receptor [46]; TGF-β and retinoic acid signaling are believed to synergize with microbial antigenic stimulation via the T-cell receptor and the chromatin-modifying activities of bacterial fermentation products, such as short chain fatty acids, to promote RORγt^+^ pTreg differentiation [41]. Our results indicate that CD103^+^ DCs have an additional, non-redundant function during the recruitment of pTregs to sites of infection, as we find that the induction of CXCR3 expression in pTregs during their priming in the LNs is highly dependent on this DC subset. The lack of CXCR3 expression, in conjunction with an impaired CXCL9/10 chemokine gradient, compromises the ability of pTregs to traffic to infected tissues. Our data are in line with two recently proposed concepts. On the one hand, there is now solid evidence for the idea that Th1-polarized settings of viral or bacterial infection promote Tregs that, like their effector T-cell counterparts, express Tbet and CXCR3 [47]; indeed, we find that *H*. *pylori* induces concomitant Th1 and Tbet^+^ pTreg responses, and that both depend on BATF3. On the other hand, co-expression of Tbet with Foxp3 is essential for Treg cells to control Th1 responses, as the depletion of Tbet-expressing Treg cells -but not of Tbet expression in Treg cells-results in severe Th1 autoimmunity [48].

Our observations together with the published data may explain the defect of BATF3^-/-^ mice in various models of peripheral and central immune tolerance. We have reported previously that BATF3^-/-^ mice, in contrast to their WT counterparts, are not protected against allergic airway inflammation upon *H*. *pylori* infection [29]; others have observed similar defects of BATF3^-/-^ mice in models of pulmonary tolerization by inhaled antigen [49], of graft-versus-host disease after allogeneic hematopoietic stem cell transplantation [50], and of presentation of Aire-induced self-antigens to developing thymocytes [51].

In summary, our results suggest that CD103^+^ DCs promote T-cell trafficking through two complementary mechanisms, i.e. by inducing the expression of CXCR3 on effector and regulatory T-cells during priming in the draining lymph nodes and by establishing a gradient of the CXCR3 ligands CXCL9 and CXCL10 in infected tissues.

## Materials and methods

### Mice

C57BL/6 and BATF3^-/-^ mice were purchased from the Jackson laboratory. CD45.1 congenic mice were obtained from a local repository. All strains were bred and maintained under specific pathogen-free conditions in accredited animal facilities at the University of Zurich.

### *H*. *pylori* infection

The *H*. *pylori* strain used in this study, PMSS1, is a clinical isolate of a patient with duodenal ulcer and the parental strain of the mouse-derivative Sydney strain 1 (SS1) [19] and was originally obtained from Adrian Lee, Univ. of New South Wales, Sydney, Australia. *H*. *pylori* was grown on horse blood agar plates and in liquid culture as described previously [19]. Cultures were routinely assessed by light microscopy for contamination, morphology, and motility. Mice were infected orally on two consecutive days with 10^8^ CFU *H*. *pylori* PMSS1 at 7 days or 6 weeks of age and analyzed at 1 or 4 months p.i. unless specified otherwise. For adoptive transfer experiments, 1 Mio splenic immunomagnetically isolated (naïve CD4 T-cell isolation kit, MagCellect, R&D Systems) naïve CD4^+^ T-cells from infected murine donors were transferred into recipient mice that had been infected for four weeks with *H*. *pylori*.

### *M*. *bovis* BCG infection

Mice were infected intraperitoneally with 5x10^6^ CFU *M*. *Bovis* BCG 1721, a derivative of *M*. *bovis* BCG Pasteur. Mice were euthanized on day 21 p.i., and spleens and livers were harvested and weighed. To assess BCG colonization, spleens and livers were individually homogenized and serial dilutions were plated on antibiotic-free Middlebrook Agar 7H10 plates (BD Biosciences), supplemented with Middlebrook OADC enrichment (BD Biosciences). Splenocytes and liver leukocyte preparations were re-stimulated *in vitro* with 5 μg/ml BCG-specific purified protein derivate (PPD) from Statens Serum Institut (Copenhagen, Denmark) and subsequently analyzed by flow cytometry for production of cytokines.

### MC38 tumor model

The murine colorectal cancer cell line MC38 is derived from C57BL/6 mice and was generously provided by Prof. Lubor Borsig (University of Zurich, Zurich, Switzerland). Cells were maintained in Dulbecco’s modified Eagle’s medium supplemented with 100U/ml penicillin/streptomycin and 10% heat-inactivated fetal calf serum (FCS) at 37°C in 5% CO_2_. 5×10^5^ MC38 cells were subcutaneously injected in both flanks of C57BL/6 WT or BATF3^*-/-*^ mice, and the tumors were analyzed after 15 days for tumor weight, volume, leukocyte infiltration and RNA expression profile. The CXCR3 blocking antibody (clone: CXCR3-173) was purchased from BioXCell (West Lebanon, NH) and administered twice weekly intraperitoneally (first dose: 500 μg/dose, all subsequent doses: 250 μg) starting on the day of tumor cell injection or *H*. *pylori* infection.

### Depletion of intestinal commensals by antibiotic treatment

For the depletion of gut commensal microbiota, animals were given ampicillin (1 g l^−1^; Sigma), vancomycin (500 mg l^−1^; Applichem), neomycin sulphate (N; 1 g l^−1^; Applichem), and metronidazole (1 g l^−1^; Sigma) in their drinking water for four weeks as described previously [52].

### Leukocyte isolation

For LP leukocyte isolation, gastrointestinal tissues were opened longitudinally, washed and cut into pieces. Pieces were incubated in Hanks' balanced salt solution with 10% FCS and 5 mM EDTA at 37°C to remove epithelial cells. Tissue was digested at 37 °C for 50 min in a shaking incubator with 15 mM HEPES, 500 U/ml of type IV collagenase (Sigma-Aldrich) and 0.05 mg ml^−1^ DNase I in supplemented RPMI-1640 medium. Cells were then layered onto a 40/80% Percoll gradient, centrifuged, and the interface was washed in PBS with 0.5% BSA. Lymph node cell suspensions were prepared by digesting the LNs in 500 U/ml of type-IV collagenase in RPMI-1640 for 15 min followed by pushing through a cell strainer using a syringe plunger. Splenocytes suspensions were prepared by pushing the spleens through a cell strainer followed by ACK red blood cell lysis. Lung cell suspensions were prepared by perfusing the lung with PBS, followed by pushing the lung through a cell strainer. Similarily, 80–100 mg tumor slices were cut into pieces and digested in type IV collagenase and 0.05 mg/ml DNase I at 37 °C for 50 min under agitation in supplemented RPMI-1640 medium, followed by pushing through a cell strainer. Liver cell suspensions were prepared by chopping the liver into pieces and digestion at 37 °C for 45 min in a shaking incubator with 500 U/ml of type IV collagenase (Sigma-Aldrich) and 0.05 mg ml−1 DNase I in supplemented RPMI-1640 medium. Cells were layered onto a 40/75% Percoll gradient, centrifuged and the interface was washed in PBS with 0.5% BSA.

### Flow cytometry, T-cell re-stimulation and cell counting

Cells were stained with a fixable viability dye and a combination of the following antibodies: anti-mouse CD45 (clone 30-F11), CD45.1 (A20), CD11c (N418), MHCII (M5/114.15.2), F4/80 (BM8), CD103 (M2E7), CD11b (M1/70), CD3e (145-2C11), CD4 (RM4-5), CD8 (53–6.7), NK1.1 (PK136), TCRβ (H57-597), CD44 (1M7), CD69 (H1.2F3), Neuropilin-1 (3E12), TIGIT (1G9), CXCR3 (CXCR3-173) and CXCL9 (MIG-2F5.5), Ki67 (16A8), CD45.1 (A20), or an IgG isotype control (all from BioLegend), as well as TIM3 (FAB1529P, R&D). Fc block (anti-CD16/CD32, Affymetrix) was included to minimize nonspecific antibody binding. For intracellular cytokine staining, the cells were incubated at 37°C for 3.5 h in complete IMDM medium containing 0.1 μM phorbol 12-myristate 13-acetate and 1 μM ionomycin with 1:1,000 brefeldin A (eBioscience) and GolgiStop solutions (BD Biosciences) at 37 °C in a humidified incubator with 5% CO2. Where indicated, CD107a (LAMP-1, Biolegend) was included during the T-cell re-stimulation. MC38 tumor-cell suspensions were re-stimulated with H-2Kb-restricted MC38 peptide (KSPWFTTL) at 37°C for 3.5 h in complete IMDM medium containing brefeldin A and GolgiStop solutions. Following surface staining, cells were fixed and permeabilized with the Cytofix/Cytoperm Fixation/Permeabilization Solution Kit (BD Biosciences) according to the manufacturer's instructions. Cells were stained for 50 min with antibodies to IL-17A (TC11-18H10.1), IFN-γ (XMG1.2) and TNF-α (MP6-XT22). For the intracellular staining of Granzyme B, cells were surface stained followed by fixation and permeabilization with the Cytofix/Cytoperm Fixation/Permeabilization Solution Kit and staining with anti-Granzyme B (QA16A02, Biolegend). For the intranuclear staining of transcription factors, cells were fixed and permeablized with the Foxp3/Transcription Factor Staining Buffer Set (eBioscience) after surface staining according to manufacturer’s instructions. Cells were stained for 50 min with antibodies to FoxP3 (FJK-16s), RORγt (B2D) from Invitrogen and Tbet (4B10) (from Biolegend). Samples were aquired on a LSRII Fortessa (BD Biosciences) and analyzed using Flowjo software.

### Quantitative PCR

RNA was isolated from scraped gastric mucosa or from FACS-sorted cells using the RNeasy Mini kit (QIAGEN) according to the manufacturer's instructions. Complementary DNA synthesis was performed using Superscript III reverse transcriptase (QIAGEN). Quantitative PCR reactions for the candidate genes were performed using TaqMan gene expression assays (Csf2 Mm01290062_m1, Cxcl9 Mm00434946_m1, Cxcl10 Mm00445235_m1, Cxcl11 Mm00444662_m1, Foxp3 Mm00475162_m1, Gzmb Mm00442837_m1, Hprt Mm03024075_m1, Ifng Mm01168134_m1, Il2 Mm00434256_m1, Il4 Mm00445259_m1, Il10 Mm01288386_m1, Il12a Mm00434169_m1, Il13 Mm00434204_m1, Il23 Mm00518984_m1, Tbx21 Mm00450960_m1, Tnf Mm00443258_m1, Rorc Mm01261022_m1; Applied Biosystems). Complementary DNA samples were analyzed in duplicate using a Light Cycler 480 detection system (Roche) and gene expression levels for each sample were normalized to HPRT expression. Mean relative gene expression was determined, and the differences were calculated using the 2ΔC(t) method.

### Statistical analysis

Statistical analysis was performed with Prism 6.0 (GraphPad Software). The nonparametric Mann-Whitney U test was used for all direct statistical comparisons between two groups. Multiple group comparisons were performed by one-way ANOVA followed by Holm-Sidak’s multiple comparisons correction. Differences were considered statistically significant when p< 0.05. * indicates p<0.05, ** p<0.01, *** p<0.001, **** p<0.0001.

### Ethics statement

All animal experimentation was reviewed and approved by the Zurich Cantonal Veterinary Office, which is the relevant body regulating animal work at the University of Zurich (licences ZH67/2012, ZH24/2013, ZH170/2014, ZH224/2014, ZH140/2017 and ZH212/2018) and adheres to the rules and regulations of the Swiss National Veterinary Office.

## Supporting information

S1 Fig The gastric lamina propria of BATF3^-/-^ mice is populated by normal numbers of macrophages and CD11b+ DCs and DCs of various subsets produce chemokines that activate CXCR3. (A-C) BATF3^-/-^ and WT mice were infected with *H*. *pylori* for one or four months and their gastric LP leukocytes were analyzed by FACS. Absolute counts of CD11b^+^ DCs, CD103^+^ CD11b^+^ DCs and macrophages in the gastric lamina propria of WT and BATF3^-/-^ mice in the steady state and at one and four months p.i. with *H*. *pylori*, as determined by multi-color flow cytometry, are shown in A. Absolute counts of IFN-γ^+^ IL-17^-^ CD4^+^ T-cells and of IL-17^+^ IFN-γ^-^ CD4^+^ T-cells are shown in B. Absolute counts of Tbet^+^ and of RORγt^+^ CD4^+^ T-cells are shown in C. (D) Representative FACS plots of Ki67 staining vs. CD4 of LP preparations of BATF3^-/-^ and WT mice, corresponding to the summary plot shown in Fig 1E. (E,F) BATF3^-/-^ and WT mice were co-housed from birth onwards and infected with *H*. *pylori* for one month; their gastric LP leukocytes were analyzed by FACS along with those prepared from uninfected co-housed controls of both genotypes. *H*. *pylori* colonization is shown in E, and absolute counts of IFN-γ^+^ IL-17^-^ CD4^+^ T-cells, of IL-17^+^ IFN-γ^-^ CD4^+^ T-cells and of TNF-α^+^ CD4^+^ T-cells are shown in F. (G) DCs of the indicated subsets were flow cytometrically sorted from LP preparations of *H*. *pylori*-infected (one month) and control mice, and subjected to qRT-PCR analysis with primers specific for CXCL9, 10 and 11. Each dot represents pooled cells sorted from 2–3 mice.
(DOCX)

S2 Fig BATF3-dependent DCs are required for Th1 but not Th17 or Th2 responses during systemic bacterial infection. (A,B) BATF3^-/-^ and WT mice were infected with *M*. *bovis* BCG. At three weeks p.i., frequencies of liver IL-17^+^ CD4^+^ T-cells were quantified by intracellular cytokine staining (A) and the indicated transcripts were analyzed by qRT-PCR of total liver RNA (B).
(DOCX)

S3 Fig BATF3-/- mice fail to recruit activated CD4^+^ and CD8^+^ T-cells to the tumor microenvironment but exhibit normal frequencies and activation of NK cells. (A-I) WT and BATF3^-/-^ mice were subcutaneously injected in both flanks with 5×10^5^ MC38 cells. Tumors were analyzed after 15 days with respect to the infiltration of various leukocyte populations, and to gene expression. Absolute numbers per mg of tumor tissue of the two indicated DC populations are shown in A. Absolute numbers per mg of tumor tissue of CD4^+^ and CD8^+^ T-cells are shown in B. The frequencies of intratumoral NK cells are shown in C. Absolute numbers per mg of tumor tissue of cytokine-expressing CD4^+^ and CD8^+^ T-cells are shown in D and E, and the activation of intratumoral NK cells, as assessed by CD69 staining is shown in F. (G) Granzyme B expression by intratumoral CD8^+^ T-cells as assessed by intracellular cytokine staining and granzyme B transcript levels as assessed by qRT-PCR of unsorted tumor tissue. (H) TNF-α transcript levels as assessed by qRT-PCR of unsorted tumor tissue. In A-G, left panel, a representative study of two independent ones is shown. In G, right panel and H, pooled data from the two studies shown in Fig 3 are shown. (I) Gating strategy for the FACS-based quantification of CXCL9-positive cells among CD11c^+^ DCs; the isotype control is shown on the left.
(DOCX)

S4 Fig The recruitment of peripherally induced Tregs to infected tissues is impaired in BATF3^-/-^ mice. (A-H) BATF3^-/-^ and WT mice were infected at six weeks of age with *H*. *pylori* for one month and their gastric lamina propria Treg compartment was analyzed by FACS relative to uninfected controls of both genotypes. Absolute counts per stomach are shown for all Foxp3^+^ Tregs in A, for neuropilin-positive tTregs in B and for neuropilin-negative pTregs in C; D and E show absolute counts of Tbet^+^ pTregs and of Tbet^+^ RORγt^+^ pTregs. The expression of TIGIT, CD44 and TIM3 is shown in neuropilin-negative pTregs in F-H. (I-M) BATF3^-/-^ and WT mice were co-housed from birth, and infected at six weeks of age with *H*. *pylori* for one month; their gastric lamina propria Treg compartment was analyzed by FACS relative to uninfected controls of both genotypes. Absolute counts per stomach are shown for the indicated Treg subsets in I-M. Horizontal lines indicate medians throughout; p-values were calculated using one-way ANOVA followed by Holm-Sidak’s multiple comparisons correction. Results in A-E are pooled from two independent studies; data in F-H are from a representative study of the two shown in A-E, and the co-housing study (I-M) was performed once.
(DOCX)

S5 Fig MLN Treg populations in *H. pylori*-infected WT and BATF3^-/-^ mice. (A-C) BATF3^-/-^ and WT mice were infected at six weeks of age with *H*. *pylori* for one month and their MLN Treg compartment was analyzed by FACS relative to uninfected controls of both genotypes. All MLNs were collected and stained for this purpose. Absolute counts in all MLNs are shown for all Foxp3^+^ Tregs in A, for neuropilin-positive tTregs in B and for neuropilin-negative pTregs in C. (D-F) BATF3^-/-^ and WT mice were co-housed from birth, but otherwise treated as described in A-C. The frequencies of the indicated Treg populations are shown. Horizontal lines indicate medians throughout; p-values were calculated using one-way ANOVA followed by Holm-Sidak’s multiple comparisons correction.
(DOCX)
