# Supplementary material for: BATF3-dependent dendritic cells drive both effector and regulatory T-cell responses in bacterially infected tissues
Source: PLoS Pathog. 2019 Jun 12;15(6):e1007866. doi: 10.1371/journal.ppat.1007866 (PMC6590837; doi:10.1371/journal.ppat.1007866)
Supplement: S1 Fig — (A-C) BATF3-/- and WT mice were infected with H. pylori for one or four months and their gastric LP leukocytes were analyzed by FACS. Absolute counts of CD11b+ DCs, CD103+ CD11b+ DCs and macrophages in the gastric lamina propria of WT and BATF3-/- mice in the steady state and at one and four months p.i. with H. pylori, as determined by multi-color flow cytometry, are shown in A. Absolute counts of IFN-γ+ IL-17- CD4+ T-cells and of IL-17+ IFN-γ- CD4+ T-cells are shown in B. Absolute counts of Tbet+ and of RORγt+ CD4+ T-cells are shown in C. (D) Representative FACS plots of Ki67 staining vs. CD4 of LP preparations of BATF3-/- and WT mice, corresponding to the summary plot shown in Fig 1E. (E,F) BATF3-/- and WT mice were co-housed from birth onwards and infected with H. pylori for one month; their gastric LP leukocytes were analyzed by FACS along with those prepared from uninfected co-housed controls of both genotypes. H. pylori colonization is shown in E, and absolute counts of IFN-γ+ IL-17- CD4+ T-cells, of IL-17+ IFN-γ- CD4+ T-cells and of TNF-α+ CD4+ T-cells are shown in F. (G) DCs of the indicated subsets were flow cytometrically sorted from LP preparations of H. pylori-infected (one month) and control mice, and subjected to qRT-PCR analysis with primers specific for CXCL9, 10 and 11. Each dot represents pooled cells sorted from 2–3 mice. (DOCX) [file ppat.1007866.s001.docx]

**Figure S1**

**Figure S1. The gastric lamina propria of BATF3^-/-^ mice is populated by normal numbers of macrophages and CD11b^+^ DCs and DCs of various subsets produce chemokines that activate CXCR3.** (A-C) BATF3^-/-^ and WT mice were infected with *H. pylori* for one or four months and their gastric LP leukocytes were analyzed by FACS. Absolute counts of CD11b^+^ DCs, CD103^+^ CD11b^+^ DCs and macrophages in the gastric lamina propria of WT and BATF3^-/-^ mice in the steady state and at one and four months p.i. with *H. pylori*, as determined by multi-color flow cytometry, are shown in A. Absolute counts of IFN-γ^+^ IL-17^-^ CD4^+^ T-cells and of IL-17^+^ IFN-γ^-^ CD4^+^ T-cells are shown in B. Absolute counts of Tbet^+^ and of RORγt^+^ CD4^+^ T-cells are shown in C. (D) Representative FACS plots of Ki67 staining vs. CD4 of LP preparations of BATF3^-/-^ and WT mice, corresponding to the summary plot shown in Figure 1E. (E,F) BATF3^-/-^ and WT mice were co-housed from birth onwards and infected with *H. pylori* for one month; their gastric LP leukocytes were analyzed by FACS along with those prepared from uninfected co-housed controls of both genotypes. *H. pylori* colonization is shown in E, and absolute counts of IFN-γ^+^ IL-17^-^ CD4^+^ T-cells, of IL-17^+^ IFN-γ^-^ CD4^+^ T-cells and of TNF-α^+^ CD4^+^ T-cells are shown in F. (G) DCs of the indicated subsets were flow cytometrically sorted from LP preparations of *H. pylori*-infected (one month) and control mice, and subjected to qRT-PCR analysis with primers specific for CXCL9, 10 and 11. Each dot represents pooled cells sorted from 2-3 mice.
