# Supplementary material for: BATF3-dependent dendritic cells drive both effector and regulatory T-cell responses in bacterially infected tissues
Source: PLoS Pathog. 2019 Jun 12;15(6):e1007866. doi: 10.1371/journal.ppat.1007866 (PMC6590837; doi:10.1371/journal.ppat.1007866)
Supplement: S2 Fig — (A,B) BATF3-/- and WT mice were infected with M. bovis BCG. At three weeks p.i., frequencies of liver IL-17+ CD4+ T-cells were quantified by intracellular cytokine staining (A) and the indicated transcripts were analyzed by qRT-PCR of total liver RNA (B). (DOCX) [file ppat.1007866.s002.docx]

**Figure S2**

**
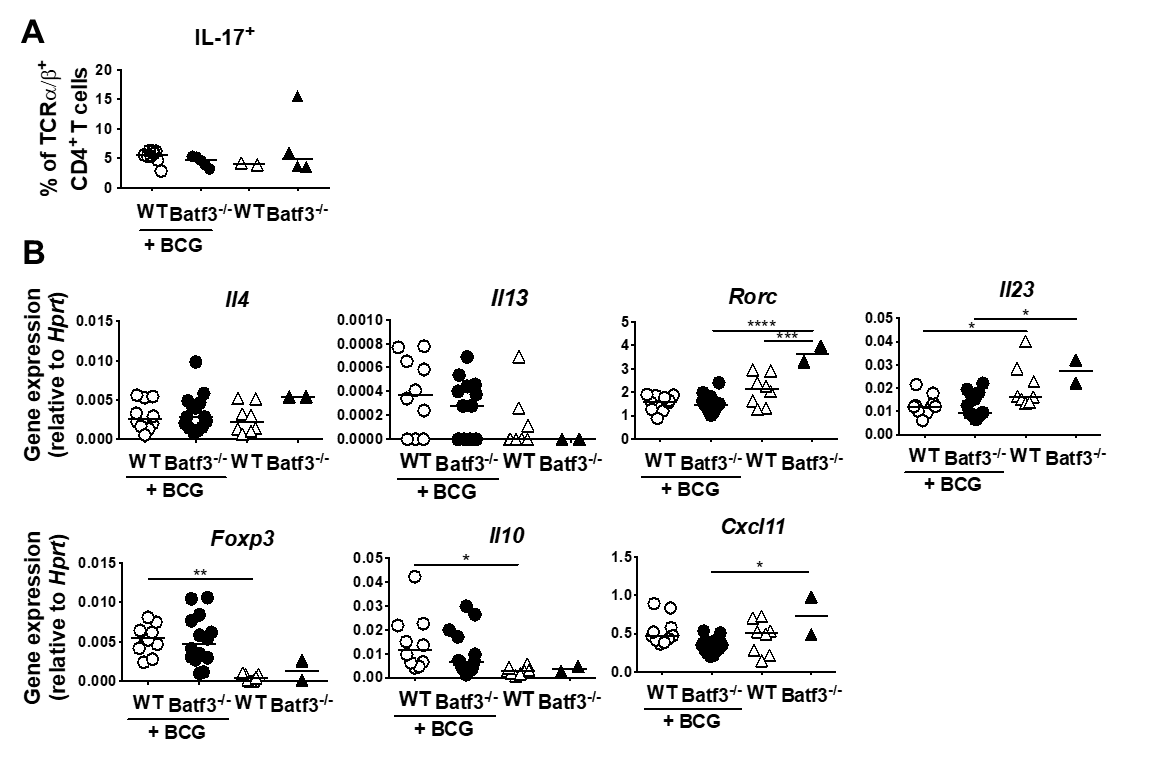
**

**Figure S2. BATF3-dependent DCs are required for Th1 but not Th17 or Th2 responses during systemic bacterial infection.** (A,B) BATF3^-/-^ and WT mice were infected with *M. bovis* BCG. At three weeks p.i., frequencies of liver IL-17^+^ CD4^+^ T-cells were quantified by intracellular cytokine staining (A) and the indicated transcripts were analyzed by qRT-PCR of total liver RNA (B).
