# Supplementary material for: BATF3-dependent dendritic cells drive both effector and regulatory T-cell responses in bacterially infected tissues
Source: PLoS Pathog. 2019 Jun 12;15(6):e1007866. doi: 10.1371/journal.ppat.1007866 (PMC6590837; doi:10.1371/journal.ppat.1007866)
Supplement: S3 Fig — (A-I) WT and BATF3-/- mice were subcutaneously injected in both flanks with 5×10^5^ MC38 cells. Tumors were analyzed after 15 days with respect to the infiltration of various leukocyte populations, and to gene expression. Absolute numbers per mg of tumor tissue of the two indicated DC populations are shown in A. Absolute numbers per mg of tumor tissue of CD4+ and CD8+ T-cells are shown in B. The frequencies of intratumoral NK cells are shown in C. Absolute numbers per mg of tumor tissue of cytokine-expressing CD4+ and CD8+ T-cells are shown in D and E, and the activation of intratumoral NK cells, as assessed by CD69 staining is shown in F. (G) Granzyme B expression by intratumoral CD8+ T-cells as assessed by intracellular cytokine staining and granzyme B transcript levels as assessed by qRT-PCR of unsorted tumor tissue. (H) TNF-α transcript levels as assessed by qRT-PCR of unsorted tumor tissue. In A-G, left panel, a representative study of two independent ones is shown. In G, right panel and H, pooled data from the two studies shown in Fig 3 are shown. (I) Gating strategy for the FACS-based quantification of CXCL9-positive cells among CD11c+ DCs; the isotype control is shown on the left. (DOCX) [file ppat.1007866.s003.docx]

**Figure S3**

**Figure S3. BATF3^-/-^ mice fail to recruit activated CD4^+^ and CD8^+^ T-cells to the tumor microenvironment but exhibit normal frequencies and activation of NK cells.** (A-I) WT and BATF3^-/-^ mice were subcutaneously injected in both flanks with 5×10^5^ MC38 cells. Tumors were analyzed after 15 days with respect to the infiltration of various leukocyte populations, and to gene expression. Absolute numbers per mg of tumor tissue of the two indicated DC populations are shown in A. Absolute numbers per mg of tumor tissue of CD4^+^ and CD8^+^ T-cells are shown in B. The frequencies of intratumoral NK cells are shown in C. Absolute numbers per mg of tumor tissue of cytokine-expressing CD4^+^ and CD8^+^ T-cells are shown in D and E, and the activation of intratumoral NK cells, as assessed by CD69 staining is shown in F. (G) Granzyme B expression by intratumoral CD8^+^ T-cells as assessed by intracellular cytokine staining and granzyme B transcript levels as assessed by qRT-PCR of unsorted tumor tissue. (H) TNF-α transcript levels as assessed by qRT-PCR of unsorted tumor tissue. In A-G, left panel, a representative study of two independent ones is shown. In G, right panel and H, pooled data from the two studies shown in Figure 3 are shown. (I) Gating strategy for the FACS-based quantification of CXCL9-positive cells among CD11c^+^ DCs; the isotype control is shown on the left.
