# Supplementary material for: BATF3-dependent dendritic cells drive both effector and regulatory T-cell responses in bacterially infected tissues
Source: PLoS Pathog. 2019 Jun 12;15(6):e1007866. doi: 10.1371/journal.ppat.1007866 (PMC6590837; doi:10.1371/journal.ppat.1007866)
Supplement: S4 Fig — (A-H) BATF3-/- and WT mice were infected at six weeks of age with H. pylori for one month and their gastric lamina propria Treg compartment was analyzed by FACS relative to uninfected controls of both genotypes. Absolute counts per stomach are shown for all Foxp3+ Tregs in A, for neuropilin-positive tTregs in B and for neuropilin-negative pTregs in C; D and E show absolute counts of Tbet+ pTregs and of Tbet+ RORγt+ pTregs. The expression of TIGIT, CD44 and TIM3 is shown in neuropilin-negative pTregs in F-H. (I-M) BATF3-/- and WT mice were co-housed from birth, and infected at six weeks of age with H. pylori for one month; their gastric lamina propria Treg compartment was analyzed by FACS relative to uninfected controls of both genotypes. Absolute counts per stomach are shown for the indicated Treg subsets in I-M. Horizontal lines indicate medians throughout; p-values were calculated using one-way ANOVA followed by Holm-Sidak’s multiple comparisons correction. Results in A-E are pooled from two independent studies; data in F-H are from a representative study of the two shown in A-E, and the co-housing study (I-M) was performed once. (DOCX) [file ppat.1007866.s004.docx]

**Figure S4**

**Figure S4. The recruitment of peripherally induced Tregs to infected tissues is impaired in BATF3^-/-^ mice.** (A-H) BATF3^-/-^ and WT mice were infected at six weeks of age with *H. pylori* for one month and their gastric lamina propria Treg compartment was analyzed by FACS relative to uninfected controls of both genotypes. Absolute counts per stomach are shown for all Foxp3^+^ Tregs in A, for neuropilin-positive tTregs in B and for neuropilin-negative pTregs in C; D and E show absolute counts of Tbet^+^ pTregs and of Tbet^+^ RORγt^+^ pTregs. The expression of TIGIT, CD44 and TIM3 is shown in neuropilin-negative pTregs in F-H. (I-M) BATF3^-/-^ and WT mice were co-housed from birth, and infected at six weeks of age with *H. pylori* for one month; their gastric lamina propria Treg compartment was analyzed by FACS relative to uninfected controls of both genotypes. Absolute counts per stomach are shown for the indicated Treg subsets in I-M. Horizontal lines indicate medians throughout; p-values were calculated using one-way ANOVA followed by Holm-Sidak’s multiple comparisons correction. Results in A-E are pooled from two independent studies; data in F-H are from a representative study of the two shown in A-E, and the co-housing study (I-M) was performed once.
