# Supplementary material for: BATF3-dependent dendritic cells drive both effector and regulatory T-cell responses in bacterially infected tissues
Source: PLoS Pathog. 2019 Jun 12;15(6):e1007866. doi: 10.1371/journal.ppat.1007866 (PMC6590837; doi:10.1371/journal.ppat.1007866)
Supplement: S5 Fig — (A-C) BATF3-/- and WT mice were infected at six weeks of age with H. pylori for one month and their MLN Treg compartment was analyzed by FACS relative to uninfected controls of both genotypes. All MLNs were collected and stained for this purpose. Absolute counts in all MLNs are shown for all Foxp3+ Tregs in A, for neuropilin-positive tTregs in B and for neuropilin-negative pTregs in C. (D-F) BATF3-/- and WT mice were co-housed from birth, but otherwise treated as described in A-C. The frequencies of the indicated Treg populations are shown. Horizontal lines indicate medians throughout; p-values were calculated using one-way ANOVA followed by Holm-Sidak’s multiple comparisons correction. (DOCX) [file ppat.1007866.s005.docx]

**Figure S5**

**Figure S5. MLN Treg populations in *H. pylori*-infected WT and BATF3^-/-^ mice.** (A-C) BATF3^-/-^ and WT mice were infected at six weeks of age with *H. pylori* for one month and their MLN Treg compartment was analyzed by FACS relative to uninfected controls of both genotypes. All MLNs were collected and stained for this purpose. Absolute counts in all MLNs are shown for all Foxp3^+^ Tregs in A, for neuropilin-positive tTregs in B and for neuropilin-negative pTregs in C. (D-F) BATF3^-/-^ and WT mice were co-housed from birth, but otherwise treated as described in A-C. The frequencies of the indicated Treg populations are shown. Horizontal lines indicate medians throughout; p-values were calculated using one-way ANOVA followed by Holm-Sidak’s multiple comparisons correction.
